# Stratification and prediction of bipolar disorders using multimodal measures

**DOI:** 10.1192/j.eurpsy.2026.10320

**Published:** 2026-07-31

**Authors:** E. Maggioni, P. Brambilla

**Affiliations:** 1 Department of Neurosciences and Mental Health, Fondazione IRCCS Ca’ Granda Ospedale Maggiore Policlinico; 2 Dept. of Electronics Information and Bioengineering, Politecnico di Milano; 3 Dept. of Pathophysiology and Transplantation, University of Milan, Milan, Italy

## Abstract

**Abstract:**

Bipolar disorder (BD) exhibits heterogeneous characteristics, frequent comorbidities, and suboptimal treatment response, negatively impacting health outcomes. Environmental and biological models are being investigated, but overall findings are fragmented. Our study proposes an innovative framework that combines multi-source and multi-site clinical, environmental and neuroimaging data with artificial intelligence tools to create and test predictive diagnostic and stratification models for BD.

Data of healthy controls (HC) and BD were analyzed, including (i) magnetic resonance imaging (MRI) from Human Connectome Project (n>1000 HC) and the STRATIBIP network (n>500 HC, n>500 BD), (ii) environmental data from Italian BD patients (n>200). Normative deep learning models of brain morphology were created using HCP and applied to STRATIBIP for anomaly detection; graph neural networks (GNNs) were applied to brain morphological networks (MBNs) to identify BD brain network markers; in Italian BD, topological data analysis (TDA) was applied to environmental data for outcome stratification.

Normative model application to STRATIBIP showed significant deviations in BD in basal ganglia and hippocampus. In STRATIBIP, BD vs. HC classification based on MBNs reached maximum accuracy with GNNs (68%), overcoming traditional ML methods. In Italian BD, TDA showed key predictive roles of (i) external environment for psychiatric comorbidities, (ii) psychiatric history for episode’s polarity.

Using advanced AI, we showed the potential of brain regions and networks as candidate BD biomarkers. We showed that brain alterations in BD are heterogeneous and partly shared with physiological variations. We found that environment plays a pivotal role in predicting BD symptomatology, underscoring the necessity to transition from unifying to multi-dimensional disease perspective.

**Disclosure of Interest:**

None Declared

